# Does economic skills obsolescence increase older workers’ absenteeism?

**DOI:** 10.5271/sjweh.4222

**Published:** 2025-05-01

**Authors:** Angela Messioui, Andries de Grip, Jos Sanders, Marion Smit

**Affiliations:** 1 Research Centre for Education and the Labour Market (ROA), Maastricht University School of Business and Economics, Maastricht, The Netherlands.; 2 HAN University of Applied Sciences, Departments Education and Development and Organisation, GL Nijmegen, The Netherlands and TNO Netherlands Organisation for Applied Scientific Research, Department Sustainable Productivity and Employability, Leiden, The Netherlands.; 3 Amsterdam University of Applied Science (AUAS), Amsterdam, The Netherlands.

**Keywords:** absence duration, absence frequency, burnout, longitudinal study, sickness, technological change, work engagement

## Abstract

**Objectives:**

This paper is the first multidisciplinary study into the impact of new skill requirements in the job on absenteeism. The aim of this study was to investigate whether economic skills obsolescence (ESO) increased both absence frequency and average duration mediated by burnout and/or work engagement.

**Methods:**

A longitudinal study was conducted on data from the Dutch Study on Transitions in Employment, Ability and Motivation (N=4493). Structural equation modelling was used to test the specific direct and indirect effects of ESO on absence frequency and average duration, followed by bootstrapping to compute the confidence intervals.

**Results:**

ESO at baseline had a positive relationship with burnout at follow-up. In turn, burnout was positively related to both absence frequency and average absence duration at follow-up. The bootstrap indirect effect test showed that ESO had a significant positive indirect effect, via burnout and (lower) work engagement, on absence frequency and average duration. Furthermore, ESO at baseline was negatively related to work engagement at follow-up. Work engagement, in turn, was negatively related to absence frequency and average duration at follow-up. The bootstrap test showed that ESO had a significant indirect effect, via work engagement, on absence frequency.

**Conclusion:**

ESO is associated with subsequent absence frequency and average duration of workers, both mediated by burnout and decreased work engagement.

Driven by accelerated technological change, the labor market faces continuous shifts in the skills required to be productive in a job (1). Particularly for older workers who have developed a skills profile during their careers, which is related to technologies that might become outdated, this creates the fear of facing skills obsolescence and losing one’s job. Changes in the world of work due to continuous shifts in the demand for skills might therefore be an important underreported cause of work-related stress (cf. 2). In the EU labor market, 29% of older workers expect several of their skills to become obsolete in the next five years (3). This risk of skills obsolescence becomes even more stressful because in many developed countries, older workers have to work longer due to pension reforms that postpone the retirement age (4). Also, various studies show that older workers face a risk of losing their employability before they are eligible to retire because of skills obsolescence, deteriorating health, and age discrimination (5).

Human capital literature distinguishes two types of skills obsolescence: technical and economic skills. Technical skills obsolescence refers to the decay and loss of workers’ skills through wear (loss of skills caused by physiological factors such as aging, illness and injuries) or atrophy because skills have been unused for extended periods of time (6, 7). Economic skills obsolescence, which is the object of this study, does not affect the skills workers possess. Instead, it refers to the obsolescence of a worker’s skills due to changing skill demands induced by technological and related organizational change of production processes by which a worker’s skills become out of date (8). As a result, workers lack the necessary skills to maintain adequate job performance and must update or upgrade their skills to remain employable (9). In practice, this economic skills obsolescence often refers to a lack of the various digital skills that are required in workers’ jobs (10) and the upgrading of job tasks due to technological change (eg 11,). Workers could (pro-actively) respond to skills obsolescence by learning new skills (8). However, older workers participate in training far less than their younger counterparts. First, they might face barriers to bridge the digital skill gaps in their job as they started their career before computerization began (12). Second, many organizations and workers have negative stereotypes of the productivity of older workers that result in “work relief policies” (eg, extra holidays, less demanding job tasks) instead of developmental opportunities such as challenging job tasks and training opportunities (13). Lastly, older workers’ training participation is declining (14). These conditions cause older workers to lack the ‘new’ knowledge they require to keep up with changes in their work, which makes them particularly vulnerable to the stress of facing economic skills obsolescence (15).

Previous studies have shown that economic skills obsolescence causes work-related stress: workers feel constant pressure to keep their skills up to date and fear a loss of their employability (16). Building on these studies, our main research question was: *Does economic skills obsolescence increase older workers’ absenteeism?* This question is highly relevant because absenteeism is a costly concern for many organizations (17, 18) and an important precedent for early labor market withdrawal.

Gommans et al (5) found that older workers who perceive they lack the necessary skills to perform adequately in their job face a higher depletion of mental and physical resources at the end of a working day, resulting in a higher need for recovery. The absenteeism literature commonly makes the distinction between absence duration and absence frequency eg (19, 20), where absence duration is often thought to be caused by health issues and absence frequency is assumed to result from the employee’s dislike of the job. However, building on Schaufeli et al (22) and Ten Brummelhuis et al (23), we expect that the distress of economic skills obsolescence could affect older workers’ absenteeism frequency as well as average duration, through burnout and work engagement. We therefore included both absence variables and the two pathways with burnout and work engagement in our analysis. Burnout refers to a combination of chronic exhaustion and a negative, cynical attitude toward work and is known as an important cause of absenteeism (22). It is most prevalent in work environments where high job demands are combined with poor or lacking resources (16). Work engagement refers to a positive work-related emotional state characterized by vitality, dedication, and absorption (22, 24). McGuinness et al (9) found that higher levels of economic skills obsolescence were associated with reduced vitality and job dissatisfaction, whereas Follmer et al (25) showed that workers facing a skills mismatch qualified this as “uncomfortable” and “frustrating”, and gradually withdrew themselves from work. As older workers have less opportunities to switch to a new job (26), their withdrawal behavior will particularly be reflected in higher absenteeism.

Many studies have examined the possible causes of absenteeism – such as physical and mental workload, illness, and worker characteristics (18). Other studies have focused on work-related factors such as having a rich job content, autonomy, social relations at work, and the status of the occupation in society (27, 28). From a multidisciplinary perspective, this study is the first to investigate the threat and stress of economic skills obsolescence as a possible driver of absenteeism. Figure 1 gives an overview of the expected relationships between economic skills obsolescence and absenteeism and underlying mechanisms.

**Figure 1:**
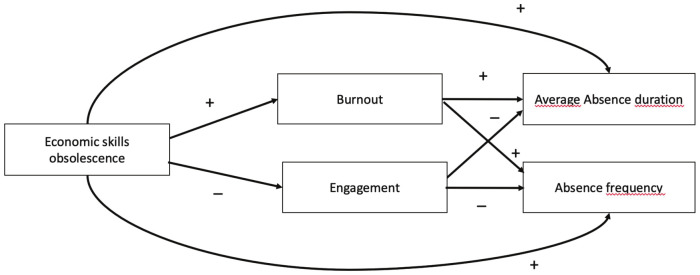
Overview of the expected relationships between economic skills obsolescence and absenteeism and the underlying mechanisms.

## Methods

### Study design

We used longitudinal panel data from the 2015, 2016, and 2017 waves of the Study on Transitions in Employment, Ability and Motivation (STREAM). A longitudinal stratified cohort study among Dutch older employees, self-employed, and non-working persons (aged 45–64 years), STREAM was designed by the Netherlands Organization for Applied Scientific Research (TNO). STREAM aims to provide better insight into factors that influence transitions in employment among ‘older’ Dutch workers and acquire knowledge on circumstances under which older individuals continue high productivity in good health. The study population of STREAM has been extensively described elsewhere (27–30). In 2015, changes were made to the STREAM questionnaire, including an adapted version of the Utrecht Burnout Scale (UBOS). Since burnout is an essential variable in our analysis, we used the fifth wave of data collection in 2015 as our baseline measurement (T1). This wave included a new sample to refill the 45–49 years category. Also, new participants were added for the age groups 50–54, 55–59, and 60–64 years to ensure enough respondents in these age groups. In 2015, in total, 16 729 participants completed the questionnaire (11 214 employees, 1150 self-employed, and 4365 non-employed).

**Figure 2:**
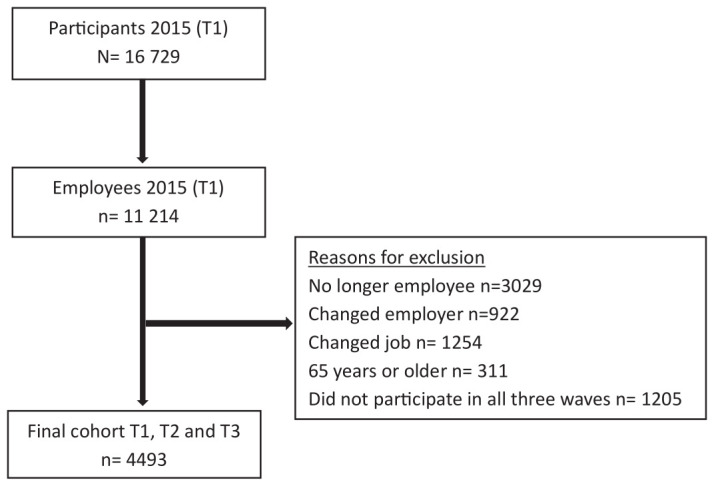
Flow chart of the exclusion procedure.

To analyze the longitudinal effects of economic skills obsolescence on absenteeism, we used data from participants in the 2015 wave (T1) and the follow-up measurements of 2016 (T2) and 2017 (T3). We excluded those who were no longer employee at T2/T3). This group consisted of non-employed persons for whom having absence days make no sense as well as self-employed participants because for them there is no formal distinction between days of absence, vacation days and (particularly for part-timers) days not working. To ascertain that economic skills obsolescence at T1 and the two absence variables at T3 refer to employment in the same job and organization, we also excluded those who changed employer or job between T1 and T3. Moreover, we excluded workers ≥65 years because 65 was at that time the mandatory retirement age in The Netherlands. Finally, we had to exclude those who did not participate in all three waves of the panel. These additional selections resulted in a final sample of 4493 workers aged 45–64 years from various industries, such as education, health care, manufacturing and services. Figure 2 describes the exclusion of the various groups of non-employees in more detail. In the supplementary material (www.sjweh.fi/article/4222), table S1 shows that the excluded participants did not differ significantly from the final sample participants in terms of economic skills obsolescence, average absence duration, absence, burnout, or work engagement at T1.

### Economic skills obsolescence

Following de Grip & van Loo (6), workers are asked to what extent they agree or disagree on a five-points scale – ranging from “strongly disagree” to “strongly agree” – with the statement “I lack ‘new’ knowledge and skills that have become important as a result of changes in my work”.

### Burnout

Burnout was measured with five items of an adapted version of the Utrecht Burn-out Scale (31). Sample items included “At the end of the working day, I feel worn out” and “I feel completely exhausted by my work.” These statements were rated on a seven-point scale from “never” to “every day” (Cronbachs α=0.91).

### Work engagement

Work engagement was measured with six items from the Utrecht Work Engagement Scale (24). Sample items included “At my job, I feel strong and vigorous” and “I am proud of the work that I do.” The answer categories ranged from “never” to “every day” on a seven-point scale (Cronbachs α=0.94).

### Covariates and controls for baseline levels of outcome variables

Workers’ age (in years), sex (male; female), education (low = primary school; medium = secondary general and vocational education; high = higher professional and academic education) were incorporated covariates. Supplementary table S2 shows the age, sex and educational composition of our sample. Moreover, we control for a worker’s health, and mental and physical workload as these variables have been associated with absenteeism in many studies (eg 22,). Health was measured with the single item “In general, would you say your health is…” rated on a five-point scale ranging from “poor” to “excellent” (32). Mental workload was measured with three items from The Netherlands Working Conditions Survey (NWCS) (eg, “Does your work require a lot of your attention?” rated on a five-point scale ranging from “never” to “always” (Cronbachs α=0.80). Physical workload was measured with five items from the Dutch Musculoskeletal Questionnaire (eg, “Does your work require a lot of force (eg, lifting, pushing, pulling)” with answer categories ranging from “never” to “always” on a five-point scale (Cronbachs α=0.86). Finally, the panel character of our data was used to control for the baseline levels of our two outcome variables, absence frequency and average duration. Supplementary table S3 gives an overview of the items from the STREAM questionnaire underlying the variables we included in this study.

### Absenteeism

Absence was assessed at T1 and at T3 with two questions: (i) “All in all, how many working days do you estimate you have been absent in the last 12 months?” and (ii) “How many times have you been absent in the last 12 months?”

On the first question, participants could respond by writing down the number of days they were absent and, for the second question, the frequency. In line with Bakker et al (17), two different measurements of absenteeism were distinguished (i): the number of absence periods in the past 12 months (*absence frequency*) and (ii) the average number of absence days in the past 12 months (*average absence duration*). Following Ten Brummelhuis et al (23), we measured average absence duration by dividing the total annual average absence duration by the number of times a worker called in sick.

Results showed that the Spearman correlation between average absence duration and absence frequency was low.

### Statistical analyses

Descriptive statistics and Spearman correlations were used to report on the general characteristics of our sample. We estimated a multiple mediation model in which we take average absence duration and absence frequency as the independent variables and burnout and work engagement as mediators. We applied the Preacher & Hayes’ (33) bootstrap method in *R* (R Core Team) (34) for multiple mediation and control variables to overcome the limitations of the stepwise approach in which the existence of indirect effects is inferred and not tested.

The Preacher & Hayes’ (33) bootstrap method enabled us to test the specific indirect effects, the total indirect effect, and the total effect. It uses four ordinary least squares (OLS) regressions to estimate the coefficients of all direct relations, followed by bootstrapping to compute the 95% confidence intervals (CI) that determine the significance of the indirect and total effects (see table 3). We estimated the model controlling for workers’ health, mental workload, physical workload, age, education and gender – which are known to relate to work engagement [eg, Schaufeli et al (22)] – as well as for average absence duration and absence frequency at baseline (2015).

**Table 3:** Indirect associations between economic skills obsolescence and absenteeism via burnout and work engagement (N=4391). Standardized regression coefficients are reported. Bootstrap sample size= 5000. [CI=confidence interval; SE=standard error.]

Bootstrap indirect effect	Via burnout				Via work engagement		
	β	SE	95% CI		β	SE	95% CI
Average absence duration	0.04	0.01	0.02–0.05		0.05	0.01	0.02–0.07
Absence frequency	0.05	0.01	0.02–0.06		0.06	0.01	0.02–0.07

To consider potential reverse causality, economic skills obsolescence was measured at T1, the two mediators at T2, and both absence variables at T3. Moreover, we adjusted for the baseline outcome variable at T1. We calculated the fit indices using R’s statistical software (R Core Team) (34). We used Tucker-Lewis index (TLI), Comparative Fit Index (CFI), and root mean square error of approximation (RMSEA) as alternative model fit measures because models with a larger sample size almost always have a statistically significant X^2^. TLI and CFI were close to 1.00 (0.99), indicating a good fit. RMSEA was 0.05 (90% CI 0.04–0.06), also indicating a good model fit [Hu & Bentler (35)].

## Results

### Descriptive information

Table 1 presents the descriptive statistics and Spearman correlations (SC). The correlation results showed that economic skills obsolescence at T1 was correlated with T3 average absence duration [SC 0.05 (P<0.001)] and T3 absence frequency [SC 0.06 (P<0.001)]. Furthermore, the results show that both mediation expectations seem plausible. There were significant correlations between economic skills obsolescence and burnout [SC 0.25 (P<0.001)] and work engagement [SC –0.20 (P<0.001)]. We also found significant correlations between burnout and both absence variables [SC 0.22 (P<0.001)] and between work engagement and both absence variables: average absence duration [SC –0.15 (P<0.001)] and absence frequency [SC –0.16 (P<0.001)]. Regarding the control variables, we found that health and workload variables were significantly correlated with burnout, work engagement, and absence frequency and duration. The correlations between age, gender, education, and both absence variables were not significant. Although we divided total annual average absence duration by the number of times a worker called in sick, we found that both at T1 and T3 average absence duration was highly correlated with absence frequency [SC 0.88 (P<0.001)] and [SC 0.95 (P<0.001), respectively)].

**Table 1:** Descriptive statistics and Spearman correlations. Cronbach’s a coefficients are shown in the diagonal. [SD=standard deviation; T=time.]

	Mean	SD	1	2	3	4	5	6	7	8	9	10	11	12	13
1. Average absence duration T3	4.82	22.97	–												
2. Absence frequency T3	1.56	6.71	0.88 ***	–											
3. Economic skills obsolescence	2.56	0.96	0.05 ***	0.06 ***	–										
4. Burnout	2.10	1.20	0.22 ***	0.22 ***	0.25 ***	0.91									
5. Work engagement	4.24	1.21	-0.15 ***	-0.16 ***	-0.20 ***	-0.45 ***	0.94								
6. Average absence duration T1	6.47	21.92	0.96 ***	0.20 ***	0.06 ***	0.25 ***	-0.17 ***	–							
7. Absence frequency T1	1.34	5.55	0.39 ***	0.88 ***	0.08 ***	0.20 ***	-0.13 ***	0.95 ***	–						
8. Health	2.67	0.83	0.20 ***	0.21 ***	0.12 ***	0.34 ***	-0.30 ***	0.25 ***	0.25 ***	–					
9. Mental workload	4.09	0.70	0.04 **	0.05 **	0.00	0.16 ***	0.09 ***	0.05 **	0.06 ***	0.01	0.80				
10. Physical workload	1.89	0.92	0.06 ***	0.05 **	0.03	0.10 ***	-0.03 *	0.06 **	0.04 *	0.10 ***	-0.10 ***	0.86			
11. Age	53.12	5.33	0.03 *	0.00	-0.05 **	-0.05 **	0.03 *	0.00	-0.01	0.04 *	0.04 *	0.01	–		
12. Education	2.08	0.77	-0.03 *	0.00	0.03 *	0.09 ***	-0.03	0.01	0.02	-0.06 ***	0.21 ***	-0.31 ***	-0.10 ***	–	
13. Gender ^a^	0.53	0.50	0.00	-0.03 *	0.03 *	0.01	-0.04 **	-0.02	-0.02	-0.03	0.06 ***	-0.02	0.11 ***	0.02	–

* P<0.05. ** P<0.01. *** P<0.001. ^a^ Gender was coded as: 0 = female, 1 = male.

Tables 2 and 3 present the direct and indirect effects – via burnout and work engagement – of economic skills obsolescence on absenteeism. The model was significant (P<0.001) and explained 10% of the variance in average absence duration and 11% in absence frequency. As expected, both burnout and work engagement mediate the relationships between economic skills obsolescence and average absence duration as well as absence frequency.

**Table 2:** Direct and indirect associations between economic skills obsolescence and absenteeism through burnout and work engagement (N=4391). Standardized regression coefficients are reported. Bootstrap sample size= 5000. [CI=confidence interval; SE=standard error.]

	Burnout				Work engagement				Average absence duration				Absence frequency		
	β	SE	95% CI		β	SE	95% CI		β	SE	95% CI		β	SE	95% CI
Economic skills obsolescence	0.12	0.02	0.09–0.15		-0.16	0.02	-0.18– -0.13		-0.01	0.02	-0.02–0.04		-0.01	0.02	-0.03–0.02
Burnout					-0.42	0.02	-0.57 – -0.50		0.15	0.01	0.11–0.18		0.14	0.01	0.12–0.16
Work engagement	-0.42	0.02	-0.57– -0.50						-0.10	0.02	-0.11– -0.02		-0.11	0.02	-0.12– -0.04
Health	0.31	0.02	0.29–0.34		-0.28	0.02	-0.30– -0.25		0.18	0.02	0.13–0.23		0.16	0.02	0.15–0.25
Mental workload	0.13	0.02	0.11–0.16		0.11	0.02	0.08–0.13		0.03	0.02	-0.01–0.09		0.02	0.03	-0.01–0.08
Physical workload	0.10	0.02	0.08–0.13		-0.02	0.02	-0.05–0.01		0.00	0.02	-0.03–0.05		0.01	0.02	-0.04–0.04
Age	-0.03	0.01	-0.03–0.00		0.00	0.01	-0.01–0.05		0.01	0.01	-0.02–0.05		0.00	0.01	-0.03–0.06
Education ^a^	0.06	0.02	0.03–0.09		-0.05	0.02	-0.08– -0.02		-0.00	0.02	-0.04–0.03		0.01	0.02	-0.06–0.02
Gender ^b^	0.01	0.03	-0.01–0.03		-0.05	0.04	-0.08 – -0.02		-0.02	0.03	-0.11– -0.03		-0.03	0.03	-0.01– -0.00
Average absence duration 2015									0.14	0.01	0.09–0.15				
Absence frequency 2015													0.09	0.01	0.07–0.12
Pseudo R^2^		0.14				0.12				0.10				0.11	

^a^ Education was coded as: 0 = lower, 1 = middle and higher. ^b^ Gender was coded as 0 = females, 1 = males.

The estimation results showed that economic skills obsolescence at baseline had a positive relationship with burnout at follow-up [β 0.12 (95% CI 0.09–0.15)]. In turn, burnout was positively related to both average absence duration [β 0.15 (95% CI 0.11–0.18)] and absence frequency [β 0.14 (95% CI 0.12–0.16)] at follow-up. The Bootstrap indirect effect test in table 3 shows the significance of this positive indirect effect of economic skills obsolescence, via burnout, on average absence duration [β 0.04 (95% CI 0.02–0.05)] and absence frequency [β 0.05 (95% CI 0.02–0.06)].

Economic skills obsolescence at baseline was negatively related to work engagement at follow-up [β -0.16 (95% CI -0.18–-0.13)]. Work engagement, in turn, was negatively related to average absence duration (β -0.10 (95% CI -0.11–-0.02) and absence frequency [β -0.11 (95% CI -0.12–-0.04)] at follow-up. Table 3 shows there was a significant indirect effect of economic skills obsolescence via work engagement on average absence duration [β 0.05 (95% CI 0.02–0.07)] and frequency [β 0.06 (95% CI 0.02–0.07)].

## Discussion

Our multidisciplinary study is the first to address the relevance of economic skills obsolescence as a stressor that induces workers’ strain to cope with the impact of rapid technological changes in their work as well as the fear for losing their job when they fail to do so. Across a three-wave longitudinal study among older workers from various industries, our findings suggested that economic skills obsolescence affected workers’ absenteeism. More specifically, we found that workers with economic skills obsolescence reported higher levels of burnout and lower levels of work engagement, which both were shown to be related to average absence duration as well as absence frequency. The latter adds to the findings of Schaufeli et al (22) and Ten Brummelhuis et al (23) that average absence duration and frequency have similar causes.

### Implications

In today’s labor market, where organizations across various industries struggle to recruit adequately skilled workers, it is essential to invest in the skills of a company’s current workforce (36). This includes older workers who suffer from economic skills obsolescence. As mentioned above, this economic skills obsolescence often refers to a lack of the digital skills that are required in workers’ jobs (10) and the upgrading of job tasks due to technological change (eg 11,).

Organizations should be aware that economic skills obsolescence is a work stressor that decreases work engagement and increases burnout and absenteeism. As technological advancements—such as the rise of AI—accelerate the pace of change in skill demands across many jobs, older workers may face increasing economic skills obsolescence in the near future, likely leading to higher rates of absenteeism. Organizations should therefore monitor economic skills obsolescence as an early-warning indicator for absenteeism. Also, lifelong learning and related pro-active HR practices should be promoted to enable older workers to keep their skills up-to-date and remain employable (37). To achieve this, organizations could help older workers to get a complete picture of their current skills and opportunities for further skill development. This will strengthen workers’ feelings of competence in performing their job and help them to prevent or overcome economic skills obsolescence, which will lower their work-related stress. Older workers would then be less inclined to call in sick when perceiving or fearing economic skills obsolescence and continue working healthy and motivated instead.

### Strengths, limitations and future research

This study is the first that studies the relationships between economic skills obsolescence and absenteeism. A major strength is the use of longitudinal data. The rich data over three data points allowed us to examine the relationships as a process that occurs over the years. The use of both absence variables enabled us to estimate an integrated model. Moreover, we controlled our estimates on average absence duration and frequency for the baseline levels of the outcome variable. While efforts have been made to test a robust model, our study has some limitations. All variables were derived from self-report measures, raising concerns about common method bias (38). However, STREAM followed recommendations for controlling the influence of common method bias, such as using validated scales (38). In addition, self-reported absenteeism is a valid method for measuring absenteeism that relates well to company-registered absence data (39), particularly because in The Netherlands absenteeism at work is clearly defined, as all days of absence are company-registered as days of sickness that can be checked by an occupational health and safety service or company doctor. Some additional limitations concerning the absence data should however be kept in mind. First, the absence measures could be intertwined (23), as shown by the high correlations. Second, also the two pathways to absenteeism we distinguish – burnout and work engagement – seem to be related since employees with higher levels of burnout also show lower work engagement. (As shown in table 1, the correlation coefficient is -0.45). A final limitation is the low percentage of variance explained by the research models, although the pseudo *R^2^* in our study corresponds with prior research on absenteeism (eg 19, 22,), reflecting that absence behavior is influenced by a wide range of factors such as physical and mental health, organizational culture, job insecurity and personal circumstances.

Future research could therefore explore other mediators between economic skills obsolescence and absence behavior, such as job insecurity and organizational support, to capture a more comprehensive picture of the pathways linking economic skills obsolescence to absenteeism. Given the scarcity of available research, it is important to follow up on the impact of economic skills obsolescence. We encourage future research to use alternative approaches, such as more in-depth case studies as well as examining the effects of economic skills obsolescence on younger workers. An interesting future research avenue could also be the study of technical skills obsolescence due to illness, aging, or non-use (6, 7) and its impact on economic skills obsolescence. This is particularly relevant for workers who have been absent from work for an extended period, who might also face economic skills obsolescence when they aim to return to work. Finally, it would be valuable to examine measures to reduce economic skills obsolescence and prevent its negative consequences.

### Concluding remarks

Based on findings from the current study, we conclude that economic skills obsolescence increases older workers’ average absence duration and frequency through increased burnout as well as decreased work engagement. These findings suggest that organizations should monitor economic skills obsolescence as an early warning indicator for absenteeism and promote lifelong learning and related pro-active HR practices that prevent economic skills obsolescence.

## Supplementary material

Supplementary material

## Data Availability

The data that support the findings of this study are available from TNO. Restrictions apply to the availability of these data, which were used under license for this study.
