# Supplementary material for: Does economic skills obsolescence increase older workers’ absenteeism?
Source: Scand J Work Environ Health. 2025 Apr 27;51(3):247–54. doi: 10.5271/sjweh.4222 (PMC12074675; doi:10.5271/sjweh.4222)
Supplement: Supplementary material [file SJWEH-51-247-S001.pdf]

# Does economic skills obsolescence increase older workers' absenteeism?[1]

*by Angela Messioui,[2] Andries de Grip, Jos Sanders, Marion Smit,*

1. Supplementary material
2. Correspondence to: Angela Messioui, Research Centre for Education and the Labour Market (ROA), Maastricht University, P.O. Box 616, 6200 MD Maastricht, The Netherlands. [E-mail: a.messioui@maastrichtuniversity.nl]

**Table S1.** *Differences in key variables at T1 of those included and excluded in the final panel*

| Variables | Final sample Mean | Excluded participants Mean | sign |
|---|---|---|---|
| Economic skill Obsolescence | 2.56 | 2.57 | . |
| Average absence duration | 4.82 | 4.80 | . |
| Absence frequency | 1.56 | 1.47 | . |
| Work engagement | 4.24 | 4.36 | . |
| Burnout | 2.10 | 2.12 | . |

**Table S2.** *Age, sex and educational composition of sample*

| Variables | | n | % |
|---|---|---|---|
| Sex | | | |
| | Male | 2398 | 53.4 |
| | Female | 2095 | 46.6 |
| Age 2015 | | | |
| | 45-49 years | 1506 | 33.5 |
| | 50-54 years | 1060 | 23.6 |
| | 55-59 years | 1204 | 26.8 |
| | 60-64 years | 722 | 16.1 |
| Age 2017 | | | |
| | 45-49 years | 884 | 19.7 |
| | 50-54 years | 1262 | 28.1 |
| | 55-59 years | 1150 | 25.6 |
| | 60-64 years | 1197 | 26.6 |
| Education | | | |
| | Lower | 1144 | 25.5 |
| | Middle | 1826 | 40.6 |
| | Higher | 1523 | 33.9 |

**Table S3.** *Items from the STREAM Questionnaire*

| Variable | Statements |
|---|---|
| Average absence duration | Quotient All in all, how many working days do you estimate you have been absent in the last 12 months? / How many times have you been absent in the last 12 months? |
| Absence frequency | How many times have you been absent in the last 12 months? |
| Economic skills obsolescence | I lack 'new' knowledge and skills that have become important because of changes in my work |
| Burnout | At the end of the working day, I feel worn out |
| | I feel completely exhausted by my work |
| | I feel emotionally drained by my work |
| | I feel exhausted when I get up in the morning and face my work |
| | It takes a lot out of me to work with people all day long |
| Work engagement | At my work, I feel bursting with energy |
| | At my job, I feel strong and vigorous |
| | I am enthusiastic about my job |
| | My job inspires me |
| | When I get up in the morning, I feel like going to work |
| | I am proud of the work that I do |
| Mental workload | Does your work require you to think very hard? |
| | Does your work require that you keep your mind on your job? |
| | Does your work require a lot of your attention? |
| Physical workload | Does your work require a lot of force (e.g., lifting, pushing, pulling)? |
| | Do your work require tools etc. causing body vibration? |
| | Do you work in uncomfortable postures? |
| | Do you stand for long periods of time? |
| | Do you kneel or squat for longer periods of time? |
| Health | In general, would you say your health is… |
